# Effect of the *MDM2* promoter polymorphisms SNP309T>G and SNP285G>C on the risk of ovarian cancer in *BRCA1* mutation carriers

**DOI:** 10.1186/1471-2407-12-454

**Published:** 2012-10-05

**Authors:** Merete Bjørnslett, Stian Knappskog, Per Eystein Lønning, Anne Dørum

**Affiliations:** 1 Department of Medical Genetics, Oslo University Hospital – The Norwegian Radium Hospital, Oslo, Norway; 2 Institute of Clinical Medicine, University of Oslo, Oslo, Norway; 3 Section of Oncology, Institute of Medicine, University of Bergen, Bergen, Norway; 4 Department of Oncology, Haukeland University Hospital, Bergen, Norway; 5 Department of Gynecological Oncology, Oslo University Hospital – The Norwegian Radium Hospital, PO Box 4953, Nydalen, N-0424, Oslo, Norway; 6 Faculty of Medicine, University of Oslo, Oslo, Norway

**Keywords:** Ovarian cancer, BRCA, MDM2 SNP285, MDM2 SNP309

## Abstract

**Background:**

While *BRCA* mutation carriers possess a 20-40% lifetime risk of developing ovarian cancer, knowledge about genetic modifying factors influencing the phenotypic expression remains obscure. We explored the distribution of the *MDM2* polymorphisms SNP309T>G and the recently discovered SNP285G>C in Norwegian patients with *BRCA* related ovarian cancer.

**Methods:**

221 *BRCA* related ovarian cancer cases (*BRCA1; n* = 161 and *BRCA2*; *n* = 60) were tested for the *MDM2* polymorphisms. Results were compared to healthy controls (*n* = 2,465).

**Results:**

The SNP309G allele was associated with elevated OR for ovarian cancer in *BRCA1* mutation carriers (SNP309TG: OR 1.53; CI 1.07-2.19; *p* = 0.020; SNP309GG: OR 1.92; CI 1.19-3.10; *p* = 0.009; SNP309TG+GG combined: OR 1.61; CI 1.15-2.27; *p* = 0.005). In contrast, the SNP285C allele reduced risk of *BRCA1* related ovarian cancer in carriers of the SNP309G allele (OR 0.50; CI 0.24-1.04; *p* = 0.057). Censoring individuals carrying the SNP285C/309G haplotype from the analysis elevated the OR related to the SNP309G allele (OR 1.73; CI 1.23-2.45; *p* = 0.002). The mean age at disease onset was 3.1 years earlier in carriers of SNP309TG+GG as compared to carriers of SNP309TT (*p* = 0.068). No such associations were found in *BRCA2* related ovarian cancer.

**Conclusions:**

Our results indicate the SNP309G allele to increase and the SNP285C allele to reduce the risk of *BRCA1* related ovarian cancer. If confirmed in independent studies, this finding may have implications to counseling and decision-making regarding risk reducing measures in *BRCA1* mutation carriers.

## Background

The prognosis of ovarian cancer is dismal with a 5 year overall survival of 40-45%, as two thirds are diagnosed with advanced disease, when the success of therapeutic modalities is very limited [1-3]. The average annual incidence rate is 10 per 100,000 women, and 8-13% carries a germline mutation in the *BRCA* genes [3-7]. Meta-analyses have revealed a wide variance in cumulative ovarian cancer risk for *BRCA* mutation carriers, on average 40% in *BRCA1* versus 11-18% in *BRCA2* mutation carriers [1,2]. *BRCA1* related ovarian cancer are diagnosed at an earlier age than sporadic ovarian cancer cases, while average age at diagnosis for *BRCA2* mutation carriers is similar to sporadic cases [2,3]. This leaves *BRCA1* mutation carriers in particular, with a difficult decision regarding risk-reducing salpingo-oophorectomy at young age, with impact on somatic, sexual and mental morbidity [8-12]. Thus, identification of additional predictive factors modifying risk of disease may have implications to genetic counseling and timing of prophylactic surgery.

Among potential risk modifying factors are SNPs altering the function of *MDM2*, the murine double-minute 2 homolog. *MDM2* encodes an ubiquitin protein ligase targeting the tumor protein p53, as well as other proteins involved in cell cycle control like the retinoblastoma-associated protein pRb [13,14]. The *BRCA* related high grade serous carcinomas are characterized by a high level of genetic instability [15]. Overexpression and/or amplification of *MDM2* have also been considered an alternative mechanism of p53 inactivation in cancers with wild-type *TP53*[16-19].

In 2004, Arnold Levine’s group published a newly discovered polymorphism in the intronic P2 promoter of *MDM2*, SNP309T>G (rs2279744; NM_002392.3:c.14+309T>G) [20]. The SNP309G allele was shown to enhance *MDM2* transcription by extending a Sp1 binding site. Further, they reported SNP309G to be associated with accelerated formation of soft tissue sarcomas in individuals carrying a *TP53* germline mutation (Li-Fraumeni syndrome) as well as in sporadic soft tissue sarcomas, lymphomas, colorectal cancer and estrogen receptor-rich breast cancer [20-22]. However, subsequent studies attempting to address the impact of SNP309 status on cancer risk and age at disease onset in various solid malignancies have produced conflicting results [23-27].

Recently, we reported a second polymorphism in the *MDM2* P2 promoter, SNP285G>C (rs117039649; NM_002392.3:c.14+285G>C), located 24 bp upstream of SNP309 [28]. Among cancer patients and controls tested (n > 7,000), the SNP285C allele was not detected in any individuals carrying the SNP309TT genotype. Thus, we found the SNP285C variant in complete linkage disequilibrium with the SNP309G allele, creating the distinct SNP285C/309G haplotype (*p* < 1.0 x 10^-10^) [28]. SNP285C antagonizes the biological effect of SNP309G by reducing the Sp1 ligand binding and reduces the risk of several cancer types [28,29]. Interestingly, the SNP285C/309G haplotype was detected in West European Caucasians, accounting for 11.7% of the SNP309G alleles, but was absent in a Chinese population. Thus, SNP285C may be a confounding factor providing ethnic disparity in evaluating potential impact of SNP309 on cancer risk [30].

In our recent study, we reported an elevated risk of sporadic ovarian cancer in Caucasians carrying the SNP309G allele [28]. In contrast, we found the SNP285C allele to reduce ovarian cancer risk by 37% in carriers of the SNP285GC/309TG genotype versus carriers of the SNP285GG/309TG genotype [28]. Here we report the distribution of the *MDM2* SNP285G>C and SNP309T>G polymorphisms in 221 Norwegian ovarian cancer patients diagnosed with germline mutations in *BRCA1* (*n* = 161) and *BRCA2* (*n* = 60). We compared the distribution of the *MDM2* polymorphisms SNP285G>C and SNP309T>G in these patients to a group of 2,465 healthy controls [28].

## Results

### Effect of *MDM2* SNP status on ovarian cancer risk in *BRCA* mutation carriers

Among the 221 mutation carriers, 161 carried a germline mutation in *BRCA1* and 60 in *BRCA2*. Distribution of the *MDM2* promoter SNPs in *BRCA* carriers and healthy controls are presented in Table 1, together with previously published data of these SNPs in sporadic ovarian cancer patients [28].

**Table 1:** **Genotypic and allelic frequencies of the *MDM2 *SNP285 and SNP309 in *BRCA *related ovarian cancer, sporadic ovarian cancer and healthy controls**

	**SNP285**	***BRCA1 *related**		***BRCA2 *related**		Sporadic disease^*^		Healthy controls^*^	
		**n**	**(%)**	**n**	**(%)**	**n**	**(%)**	**n**	**(%)**
SNP285	GG	153	(95.0)	58	(96.7)	1260	(93.7)	2 274	(92.3)
	GC	8	(5.0)	2	(3.3)	82	(6.1)	183	(7.4)
	CC	0	(0.0)	0	(0.0)	3	(0.2)	8	(0.3)
	Total	161	(100)	60	(100)	1 345	(100)	2 465	(100)
	G	314	(97.5)	118	(98.3)	2 602	(96.7)	4 731	(96.0)
	C ^**^	8	(2.5)	2	(1.7)	88	(3.3)	199	(4.0)
SNP309	TT	52	(32.3)	22	(36.7)	515	(38.3)	1 072	(43.5)
	TG	81	(50.3)	32	(53.3)	661	(49.1)	1 093	(44.3)
	GG	28	(17.4)	6	(10.0)	169	(12.6)	300	(12.2)
	Total	161	(100)	60	(100)	1 345	(100)	2 465	(100)
	T	185	(57.5)	76	(63.3)	1 691	(62.9)	3 237	(65.7)
	G ^**^	137	(42.5)	44	(36.7)	999	(37.1)	1 693	(34.3)

^*^ Previously published data [28].

^**^ Minor allele.

For SNP309, we found the frequency of the minor allele, SNP309G, to be significantly higher in *BRCA1* mutation carriers (42.5%) than in healthy controls (34.3%; *p* = 0.003). Notably, censoring individuals harboring the SNP285C allele (previously shown to counteract the effect of SNP309G) strengthened this association (*p* < 0.001). No such difference was observed for *BRCA2* mutation carriers (*p* > 0.5).

**Figure 1:**
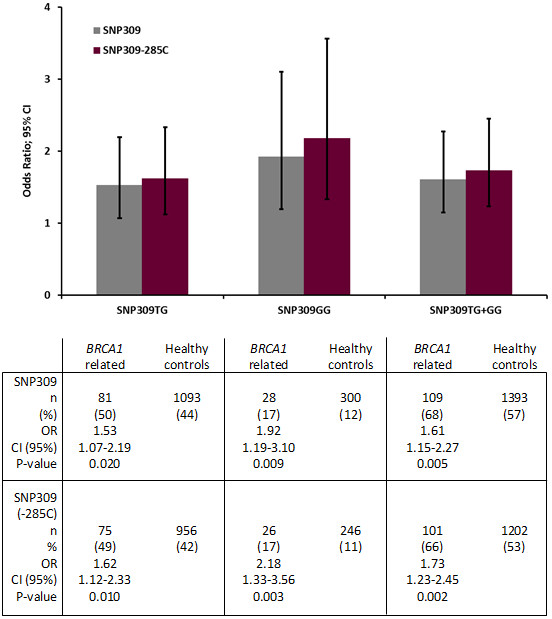
**Impact of the *MDM2 *SNP309G and SNP285C on *BRCA1 *related ovarian cancer versus healthy controls.** Bars indicate odds ratio (OR) for ovarian cancer depending on *MDM2* SNP status, error bars indicate 95% CI and *p*-values are calculated by Fisher exact test.

In *BRCA1* mutation carriers we found an elevated OR for ovarian cancer related to the SNP309TG and SNP309GG genotypes (SNP309TG; OR 1.53; CI 1.07-2.19; *p* = 0.020; SNP309GG; OR 1.92; CI 1.19-3.10; *p* = 0.009; SNP309TG + GG combined: OR 1.61; CI 1.15-2.27; *p* = 0.005) (Figure 1). Censoring individuals harboring the SNP285C allele strengthened the association between SNP309G status and increased ovarian cancer risk (SNP309TG: 1.62; CI 1.12-2.33; *p* = 0.010; SNP309GG: OR 2.18; CI 1.33-3.56; *p* = 0.003; SNP309TG + GG combined: OR 1.73; CI 1.23-2.45; *p* = 0.002).

Regarding SNP285 status, the SNP285C/309G haplotype was detected in 8 out of 161 (5%) individuals carrying a *BRCA1* mutation and 2 out of 60 (3.3%) with a *BRCA2* mutation (Table 1). Although numbers are small, the frequency of the SNP285C/309G haplotype among carriers of the SNP309G was lower in *BRCA1* mutation carriers as compared to the frequency in healthy individuals (OR 0.50; CI 0.24-1.04; *p* = 0.057). The minor allele frequency in this subgroup being 3.7% for *BRCA1* mutation carriers and 7.1% for controls (*p* = 0.051).

### Effect of *MDM2* SNP status on age at ovarian cancer onset

**Figure 2:**
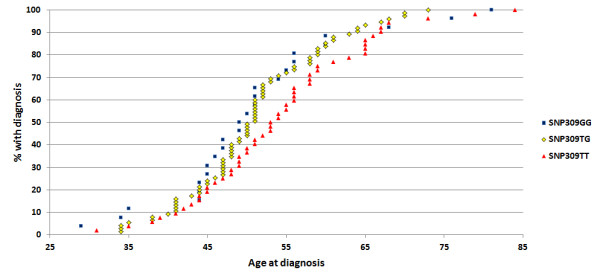
**Impact of the *MDM2 *SNP309 genotypes on age at ovarian cancer diagnosis in *BRCA1 *mutation carriers.** The cumulative percentage of individuals with the SNP309TT genotype (red triangles), the SNP309TG genotype (yellow diamonds) and the SNP309GG genotype (blue squares) plotted against age at ovarian cancer diagnosis. The SNP285C/309G haplotype is censored in the dataset.

We further examined the effect of *MDM2* SNP status with respect to age at onset of ovarian cancer in *BRCA* mutation carriers. The average age at diagnosis in patients harboring the SNP309 genotypes TT, TG and GG were 54.2, 51.2 and 51.8 years, respectively. When censoring patients harboring the SNP285C/309G haplotype, the mean age at onset for the TT, TG and GG genotypes were 54.2, 51.1 and 51.0 years, respectively. Thus, carriers of the SNP309TG and GG genotypes were 3.1 years younger as compared to carriers of the SNP309TT genotype (*p* = 0.068; Figure 2). The mean age at onset in *BRCA2* mutation carriers did not differ from sporadic ovarian cancer cases (59.6 versus 61.2 years), and we observed no differences in age at onset related to SNP309 status.

## Discussion

Currently, little is known about factors acting as genetic modifiers to ovarian cancer tumorigenesis. While several studies have explored the effect of genetic variants on cancer risk in *BRCA* mutation carriers [31-33], no distinct genetic variant that strongly predicts cancer risk in these subgroups have been identified.

Studies addressing the impact of the *MDM2* SNP309 status on risk of tumor development and age at onset in different types of cancers have provided conflicting results, with a trend for positive associations in Asians but a lack of correlation in Caucasians [23,25-27]. While the *MDM2* SNP309G is an ancient polymorphism detected in all ethnic groups examined so far, its frequency display distinct ethnic variation [26], accounting for 12.4% of the alleles in African Americans [32] contrasting about 34% in Caucasians [28,34] and 50-55% in Asians [30-37]. In contrast, SNP285C is a more recent polymorphism arising on the SNP309G allele and with a distribution so far restricted to Caucasian populations [28,30]. Previously, we found the SNP285C/309G haplotype to account for 11.7% of all the SNP309G alleles in Western Europeans (British, Dutch and Norwegian populations) but in less than 2% of Finns and absent in healthy Chinese individuals [28]. In general, this distribution has been confirmed by The 1000 Genomes Project Consortium [38]. Thus, we hypothesize that the occurrence of the SNP285C/309G haplotype may have contributed to the conflicting results from studies addressing the impact of SNP309 status on ovarian cancer risk in Caucasian populations.

Only a few studies have addressed the risk of ovarian cancer with respect to SNP309 status. Three studies (two Caucasian populations and one Japanese) reported no association between SNP309G status and elevated risk of ovarian cancer [37,39,40]. In contrast, the SNP309G allele was found to be a potential protective factor, associated with a reduced risk of ovarian cancer, in a Chinese population [36]. However, these studies included a limited numbers of patients (302 or less) without defined *BRCA* mutation status. In our previous study on Caucasians evaluating nearly 2,000 sporadic ovarian cancer cases and > 3,500 healthy controls, we found an elevated risk of ovarian cancer in carriers of the SNP309TG and SNP309GG genotypes [28]. However, we found a reduced cancer risk in carriers of the SNP309TG genotype harboring the SNP285C/309G haplotype.

To the best of our knowledge, only two studies have addressed the impact of SNP309 on ovarian cancer risk in *BRCA* mutation carriers. Yarden et al. [41] found the SNP309GG genotype to be significantly associated with risk of *BRCA1* related ovarian cancer in Ashkenazi Jews diagnosed before 51 years of age, while Copson et al. [42] detected a non-significant trend for increased incidence of ovarian cancer in British *BRCA1* mutation carriers harboring the SNP309GG genotype. While the distribution of the SNP285C allele in Ashkenazi Jews is unknown, it occurs in the British population [28], thus, it may have influenced the result reported by Copson et al. [42], partly masking an effect of the SNP309G status on ovarian cancer risk in this population.

Here, we report an elevated OR for ovarian cancer in *BRCA1* mutation carriers harboring a *MDM2* SNP309TG or SNP309GG genotype. While the exact OR values presented should be interpreted carefully due to a limited number of observations, the results indicate that SNP309 status may influence ovarian cancer risk with OR to a similar extent in *BRCA1* mutation carriers as observed in sporadic ovarian cancer [28].

While the SNP285C/309G haplotype was observed in 13.1% of the SNP309G allele carriers in healthy controls [28], the percentage in *BRCA1* mutation carriers was only 7.3% (OR 0.50; CI 0.24-1.04). The lower incidence of the SNP285C/309G haplotype found in *BRCA1* mutation carriers with ovarian cancer may indicate a risk reducing effect of the SNP285C allele. However, this finding needs confirmation by independent studies.

A key feature in *BRCA* related breast and ovarian cancer is earlier age at onset as compared to sporadic disease, with the most distinct differences related to *BRCA1* mutations. When censoring patients harboring the SNP285C/309G haplotype from our analyses, we found *BRCA1* related cancer cases carrying the SNP309TG and SNP309GG genotypes to be on average 3.1 year younger at time of diagnoses as compared to carriers of the SNP309TT genotype. Although this difference was of borderline statistical significance, it supports the finding that the SNP309G allele promotes ovarian tumorigenesis in *BRCA1* mutation carriers. If confirmed in other studies, this information may be useful for timing of risk reducing surgery in healthy *BRCA1* mutation carriers, as the age at onset in index cancer cases has been found to predict individual risk [1].

## Conclusions

In conclusion, we found the *MDM2* SNP309 to increase and SNP285C to reduce the risk of ovarian cancer in *BRCA1* related ovarian cancer. These findings may have potential implications to genetic counseling and prophylactic strategies in *BRCA1* mutation carriers.

## Methods

### Ovarian cancer patients

In Norway, ovarian cancer treatment is centralized, and The Norwegian Radium Hospital, covers 50% of the population. A total of 221 *BRCA* mutation carriers were identified among 1,566 patients diagnosed and treated for invasive epithelial ovarian cancer; 161 carrying a mutation in *BRCA1* and 60 in *BRCA2*. All patients were offered counseling and genetic testing irrespective of family history of cancer, and participants signed a written informed consent before testing. Pathology reports were reviewed in all cases. Family members of mutation carriers were offered genetic counseling, testing and medical follow-up.

### Controls

The 1,345 sporadic ovarian cancer patients (mutation carriers excluded) and the 2,465 healthy controls used for comparisons are previously described [28].

### *BRCA* mutation analysis

All included patients were subjected to genetic testing in the *BRCA* genes. Lymphocyte DNA was prepared from peripheral blood by standard procedures (MagAttract DNA Blood M48 Kit, Qiagen), amplified by polymerase chain reaction (HotMaster Taq DNA Polymerase, 5 PRIME) and subjected to subsequent analysis. Mutation analysis was performed with the application of different screening techniques (fragment length analysis with fluorescent primers, MLPA analysis (SALSA MLPA kits, MRC Holland) and direct sequencing (BigDye Terminator Cycling Sequencing Kit, Applied Biosystems) to detect genetic variations in the *BRCA* genes. All detected mutations were confirmed by independent DNA extractions, PCRs and sequencing reactions.

### *MDM2* promoter genotyping

A region of the *MDM2* P2 promoter covering SNP285 and SNP309 was amplified by PCR and sequenced as previously described [28].

### Statistical analysis

Statistical analyses were performed using the SPSS software package (version 15.0.1). Differences regarding SNP frequency were evaluated by Chi square and Fisher exact tests, and *MDM2* SNP distribution among ovarian cancer patients and healthy controls presented as odds ratio (OR). Differences in age at onset were evaluated by Kruskal-Wallis and Mann–Whitney rank tests. All *p*-values are given as two-sided and confidence intervals (CI) for odds ratios are given as 95%.

## Competing interests

The authors declare that they have no competing interests.

## Authors’ contributions

MB performed the genetic testing in the *BRCA* genes and parts of the *MDM2* SNP testing, audited the clinical data together with AD and wrote the paper; SK performed the majority of the *MDM2* SNP testing, performed the statistical analysis and co-authored the paper; PEL supervised data analysis and co-authored the paper; AD audited the clinical data and co-authored the paper. All authors read and approved the final manuscript.

## Pre-publication history

The pre-publication history for this paper can be accessed here:

http://www.biomedcentral.com/1471-2407/12/454/prepub
